# Surveying family access: kangaroo mother care and breastfeeding policies across NICUs in Italy

**DOI:** 10.1186/s13052-021-01164-8

**Published:** 2021-12-02

**Authors:** Claudia Artese, Fabrizio Ferrari, Silvia Perugi, Paola Cavicchioli, Giuseppe Paterlini, Fabio Mosca, the Developmental Care Study Group of Italian Society and Neonatology, Natascia Bertoncelli, Valeria Chiandotto, Paterizia Strola, Natascia Simeoni, Guido Calciolari, Grazia Colombo, Serena Rovei, Immacolata Arenga, Elena Arpi, Rosario Montirosso

**Affiliations:** 1 Neonatology and Neonatal Intensive Care Unit, Careggi University Hospital, Florence, Italy; 2 Neonatology and Neonatal Intensive Care Unit, University of Modena and Reggio Emilia Hospital, Via del Pozzo 71, 41125 Modena, Italy; 3 Neonatal Intensive Care Unit, Hospital “Dell’Angelo” Venezia Mestre, Venice, Italy; 4 Department of Mother’s and Child’ Health, Neonatology and Neonatal Intensive Care Unit, Poliambulanza Foundation Hospital Institute, Brescia, Italy; 5 Neonatology and Neonatal Intensive Care Unit, Department of Clinical Sciences and Community Health, University of Milan, IRCCS Cà Granda Ospedale Maggiore Foundation, University Hospital, Milan, Italy

**Keywords:** Developmental care, Parental access, Premature newborn, Breastfeeding, Skin-to-skin contact

## Abstract

**Background:**

Studies on the application of developmental care initiatives in Italian NICUs are rather scarce. We aimed to assess parental access to the NICUs and facilities offered to the family members and to test “the state of art” regarding kangaroo mother care (KMC) and breastfeeding policies in level III Italian NICUs.

**Methods:**

A questionnaire both in paper and in electronic format was sent to all 106 Italian level III NICUs; 86 NICUs (i.e., 80% of NICUs) were completed and returned.

The collected data were analysed. In addition, a comparison between the 2017 survey results and those of two previous surveys conducted from 2001 to 2006 was performed.

**Results:**

In total, 53 NICUs (62%) reported 24-h open access for both parents (vs. 35% in 2001 and 32% in 2006). Parents were requested to temporarily leave the unit during shift changes, emergencies and medical rounds in 55 NICUs (64%). Some parental amenities, such as an armchair next to the crib (81 units (94%)), a room for pumping milk and a waiting room, were common, but others, such as family rooms (19 units (22%)) and adjoining accommodation (30 units (35%)), were not. KMC was practised in 81 (94%) units, but in 72 (62%), i.e., the majority of units, KMC was limited to specific times. In 11 (13%) NICUs, KMC was not offered to the father. The average duration of a KMC session, based on unit staff estimation, was longer in 24-h access NICUs than in limited-access NICUs. KMC documentation in medical records was reported in only 59% of questionnaires. Breastfeeding was successful in a small proportion of preterm infants staying in the NICU.

**Conclusion:**

The number of 24-h access NICUs doubled over a period of 13 years. Some basic family facilities, such as a dedicated kitchen, rooms with dedicated beds and showers for the parents, remain uncommon. KMC and breastfeeding have become routine practices; however, the frequency and duration of KMC sessions reported by NICU professionals still do not meet the WHO recommendations.

**Supplementary Information:**

The online version contains supplementary material available at 10.1186/s13052-021-01164-8.

## Background

Growing data evidence [1–3] has demonstrated that the separation between parents and preterm infants is one of the most stressful aspects during neonatal intensive care unit (NICU) stays, both for parents and newborns. However, parental closeness to their baby promotes neonatal wellbeing, such as through the stabilization of cardiorespiratory functions, and leads to better bearing of the pain and stress related to the disturbing stimuli present in the NICU environment [1]. For parents to become coregulators and facilitators of neonatal development, their physical presence is essential. Family-centred care reduces morbidity and improves the neurocognitive development of preterm infants [1, 2, 4–6]. For these reasons, various authors and the World Health Organization (WHO) consider open access to the unit, kangaroo mother care (KMC) and breastfeeding of paramount importance [1, 4, 6, 7]. Keeping in mind these reasons, the Neonatal Developmental Care (NDC) study group of the Italian Society of Neonatology (SIN) has long aimed to grant parents open access to the NICU 24 h a day.

To achieve this open parental access, the WHO [7] recommends increased training for staff and the implementation of protocols and programmes to educate parents on the most effective practices for promoting developmental care (DC) and therein empower them to become primary caregivers.

A few of the studies dedicated to DC in Italian NICUs were performed during the last two decades [8–13], but some of them were focused on specific aspects of the DC theme. The 2009 de Vonderweid U. and Leonessa M [9] study was very brief and general and reported open NICU access to mothers in only 29% of Italian NICUs; the questionnaire used in the study was not reported. A European study by Greisen et al. (2009) [8] compared parental involvement and KMC in eight European countries. The study, conducted between 2004 and 2006 by a European network sponsored by the European Science Foundation [8], was based on a questionnaire on early DC practises that was mailed to 362 units in eight European countries. Of these NICUs, 78% responded, but only 175 NICUs, caring for at least 50 very-low-birth-weight (VLBW) infants every year, were considered. The study demonstrated that in northern European countries (Sweden, Denmark, the UK, the Netherlands, Belgium), both parents had open, unmitigated access to the NICU at any time. French NICUs offered an intermediate degree of access. Finally, in Spain and Italy, unrestricted NICU access was granted in only less than one-third of NICUs. Moreover, in 70% of Italian NICUs, the mother was allowed to stay in the ward only during open hours, and additional restrictions were added during medical rounds, shift changes, and emergencies. A study by Montirosso et al. (2014) [11], which was a study limited to few Italian NICUs (not a national survey) focused on the relationship between variations in DC in NICUs and the neurobehavioural development of preterm infants. A 2012 survey [12] showed that restrictive parental access policies were in place in 80% of NICUs, which was the highest amongst other countries such as Spain (73%); France (41%); and Sweden, Denmark, the UK, the Netherlands and Belgium (all between 0 and 10%). Artese et al. (2020, 15), using the same questionnaire as that used in this study, investigated barriers and facilitators to KMC on the basis of a multiple regression model to see which factors play a relevant role in predicting KMC implementation. They found that structural factors (e.g., adequate space and facilities) can support families in providing KMC, but more interestingly, they found that medical record documentation appears critical for improving the practice of KMC.

In the last few years, DC has been promoted through several residential courses in single units and through local regional NDC conferences in many Italian regions. The programme of annual national SIN meetings includes one major lecture on DC every year. Recommendations on parental access to the NICUs, KMC and breastfeeding were published [6] and distributed to all NICUs in 2017 and are now under revision for the new updated release.

To obtain an overall up-to-date view of DC through Italian NICUs and to avoid the limitations that emerged in the aforementioned studies, the board of the SIN decided to adopt a system for the periodic monitoring of changes in DC throughout the country, promoting periodic surveys.

To this effect, the present survey has two aims: first, to evaluate NICU parental access and the facilities offered to the parents, and second, to test “the state of art” of the KMC and breastfeeding policies adopted in individual Italian NICUs.

## Methods

### Participants and procedures

A multicentre descriptive observational survey of Italian NICUs hospitalizing infants less than or equal to 32 weeks of gestational age (GA) was conducted from June 2017 to January 2018.

### Survey development

The questionnaire used in our research is inserted in the section “supplemental material”; it was designed by a multidisciplinary panel of experts from the NDC study group, which consisted of six neonatologists with more than 15 years of clinical experience in DC practices in NICU DC (GP, SP, PC, GC, FM, FF, VC; one of them is Newborn Individualized Developmental Care and Assessment Program (NIDCAP) trained and the director of the Italian NIDCAP of Modena (FF)); three NIDCAP-trained nurses (NS, SR, IA; one of them, NS, is also a trainer at the Rimini NIDCAP centre); 3 physical therapists (PS, CA, NB; one of them, NB, is an NIDCAP trainer at the Modena NIDCAP centre (NB)); two psychologists (RM, EA), and one sociologist (GC). These experts identified the most significant items for an investigation of the practices in support of DC, KMC and breastfeeding. In the study by de Vonderweid and Leonessa [9], the survey questionnaire was not published, which made it impossible to perform a comparison of the present survey with the previous survey. The questionnaire (see appendix**)** was sent to all Italian NICUs both in paper and in electronic formats to facilitate online completion.

### Questionnaire design

The questionnaire was structured in four specific sections investigating general characteristics of the NICUs, parental access and attitudes towards parents, KMC, and support for breastfeeding.

### General characteristics of NICUs

To obtain information on the number of beds in the NICU and the postintensive care area, the director and the nursing coordinator completed the questionnaires. The presence of NICU consultants, such as psychologists, physiotherapists and child neurologists, and the way they were involved were also explored.

### Parental access and NICU staff attitudes towards parents

The questions concerned parental access to the ward, specifying whether it was open or time limited (maximum hours) for mothers and fathers, whether both parents and relatives were allowed to enter and stay in the NICU, and whether parents had to leave the unit during medical examinations and/or emergencies. In addition, the facilities for parents were investigated, such as a chair or armchair close to the child’s crib, a room for pumping milk, a family room, a reading room, a dedicated kitchen, a bed inside the ward, an adjoining accommodation within the hospital, access to the hospital canteen, a waiting room and whether periodic meetings (monthly, fortnightly) with parents were offered.

### KMC

We investigated whether KMC was practised in the NICU by either parent with or without time limits, specifying the duration of the single session and the daily duration of KMC, from what GA it was usually proposed, whether it was practised in all NICU spaces, whether it was proposed more than once a day, whether it was proposed for twins, whether it was practised when a newborn was receiving ventilatory support, whether it was practised when a newborn had a central catheter, whether an early approach to the breast was proposed during KMC, whether the beginning and the end of the KMC session were recorded in the medical files, when KMC was discontinued, whether there were written unit-specific protocols or recommendations, and whether KMC training had occurred in the department in the last 3 years.

### Support for breastfeeding

The following questions regarding support for breastfeeding were included in the questionnaire: Was breastfeeding an option for mothers in the ward? Where was breastfeeding permitted in the NICU (e.g., in all inpatient rooms of the NICU, in the post-NICU area, in dedicated spaces)? How often was breastfeeding permitted (often, rarely)? Were there restrictions on the use of fresh breast milk? Was equipment for simultaneous bilateral breast milk expression available? Was expression next to the baby recommended? Was there a milk bank in the NICU and a room where mothers could express their milk? Were there breast pumps in the NICU?

### Participants and procedures

A NICU professional responsible for the distribution and collection of the questionnaires was identified in each region; he or she was available to the NICU study sites to illustrate the aim of the study and to answer questions individual units may have had. An initial letter from the NDC study group accompanied the questionnaire, which was designed for self-administration. The final page of each questionnaire was for reporting the location of the investigated NICU and the signatures of the contact person, the director of the unit and the head nurse. A total of 107 questionnaires were sent to NICUs across all Italian regions, and 86 (80%) were returned by email or mail with all four sections completed.

### Statistical analysis

Rather than sampling subjects (patients or professionals), this study was designed using “purposive sampling” [9]: we proposed the questionnaire directly to the directors of the NICUs caring for newborn infants under 32 weeks GA. Therefore, sample size and statistical power determinations are not applicable for this descriptive observational study. Although the generalization of the results is limited to the sample in question, the exploratory nature of this study made it possible to capture a wide array of variables through this ad hoc questionnaire. Descriptive statistics were performed on all evaluated variables, including mean and standard deviations for continuous/scalar variables and frequencies and percentages for categorical variables. To better assess how parental access was related to the duration of KMC, NICUs with open access lasting more than 10 h were distinguished from those with access limited to less than 10 h per day. One-case nonparametric analysis was used to compare key variables obtained by the centres with limited opening hours. The duration of the KMC was estimated from the unit staff statement, not the result of recording the single KMC sessions in the medical records.

## Results

Our survey involved 86 (80%) Italian NICUs, including 43 (50%) centres from the northern regions, 19 (22%) centres from the central regions, and 24 (28%) centres from the southern regions.

### Access to the ward

One-case nonparametric analysis of key variables demonstrated that the distribution of open access to parents significantly (*p* < 0.05) varied across centres in Italy.

In total, 53 wards (62%) allowed both parents open 24-h access to the unit, while 33 (38%) centres reported access with some degree of time constraints. Of these, 9 (27%) allowed daily access for more than 10 h and 24 (73%) allowed access for less than 10 h, with an average time per day of 4 h (Table 1). During emergencies, shift changes and/or medical rounds, parents were asked to temporarily leave the unit in the majority, i.e., 55 (64%) of the units. Regarding ward access for nonparent relatives (84/86 units), in general, only grandparents and siblings were allowed, and only in certain time windows, in nearly half of the units (44 units corresponding to 51%); however, ward access to nonparent relatives was completely restricted in 38 (44%) wards, and 24-h open access was available in only two units.

**Table 1:** Access of parents and nonparental relatives to the NICU. In numbers with decimals, the decimal were avoided and the numbers were rounded to the nearest full number

***Access to the ward***	***Number and percentage of NICUs***
24-h open access for both parents	53 …, (62%)
Limited access:	33 …, (38%)
< 10 h	24 …., (73%)
> 10 h	9 …., (27%)
Parents asked to leave during clinical activities*	55 …., (64%)
Access for nonparental relatives (84/86 responders):	
24-h open access	2 (2%)
Allowed for a limited time	44 (53%)
Never allowed	38 (45%)

*shift change for the nurses, emergencies, medical rounds

### Parent facilities

In the majority of units, parents were provided an armchair next to the child’s crib in 81 (94%) of units, a room for pumping milk in 79 units (92%), a waiting room in 65 wards (80%), and access to the hospital canteen in 59 units (73%). In contrast, other basic facilities were uncommon: family rooms were available in only 19 units (22%), a reading room in only 25 units (29%), a dedicated kitchen in only 20 units (23%), and an adjoining accommodation in only 30 units (35%) (see Table 2 basic parent facilities in the NICU).

**Table 2:** Basic parent facilities in the NICU

***Type of facilities***	***Number and percentage of NICUs***
Armchair close to the crib	81 (94%)
Room for pumping milk	79 (92%)
Waiting room	65 (76%)
Access to hospital canteen	59 (79%)
Reading room	25 (29%)
Dedicated kitchen	20 (23%)
Family room	19 (22%)
Adjoining accommodation	30 (35%)

### Characteristics of KMC policies

KMC was offered in 81 units (94%), but in the majority of them (50 units (62%)), KMC was limited to a specific time of day, and in 11 (13%) of the wards, fathers were not involved. KMC was offered more than once per day in 54 units (67%). Regarding the relationship between KMC and weeks postmenstrual age (PMA), KMC was offered in 28 units (35%) at less than 29 weeks PMA, in 18 (22%) of the units between 29 and 30 weeks PMA and in 14 units (17%) at more than 30 weeks PMA. Notably, 21 units (26%) did not answer the question or simply made annotations such as “when the baby is stable”. Moreover, 70 units (86%) reported daily KMC with twins. KMC was routinely offered to preterm newborn infants on respiratory support in 58 units (72%) but in only 30 units (37%) when the infant was on mechanical ventilation, in 68 units (84%) during continuous positive airway pressure (CPAP) and high-flow nasal cannula (HFNC), and in 73 units (90%) during oxygen supplementation.

#### KMC discontinuation policies (80/81 responders)

In 46 units (57%), KMC was usually discontinued at the time of discharge from the hospital; in 10 units (13%), KMC was discontinued the beginning of full oral feeding; and in the other 24 units (30%), KMC was discontinued at the transition from intensive to postintensive care. In the presence of a central venous and/or umbilical catheter, KMC was routinely practised in 56 (68%) NICUs. Importantly, in 48 (59%) of the units, initiatives to perform KMC were recorded in the medical records. A written protocol for KMC was reported by 46 (57%) neonatal units. Finally, only 35 (43%) neonatal units reported that specific KMC training had occurred in the last 3 years. In Table 3 the characteristics of KMC facilities are mentioned.

**Table 3:** Characteristics of KMC policies

***Characteristics***	***Number and percentage of NICUs***
24 h per day	31 …. (38%)
The father never does the KMC	11 …. (13%)
Repeated more than once a day	54 …. (67%)
KMC offered from (PMA in weeks)	
< 29	28 …. (35%)
29–30	18 …. (22%)
> 30	14 …. (17%)
Others^a^	21 …. (26%)
KMC in twins	70 …. (86%)
KMC during respiratory support	
MV	30 …. (37%)
NIV	68 …. (84%)
Oxygen supply	73 …. (90%)
KMC discontinued (80/81)	
At the time of transfer to the postintensive care unit	24 …. (30%)
At the beginning of full oral feeding	10 …. (13%)
At discharge	46 …. (57%)
KMC with central lines	56 …. (69%)
Initiatives to perform KMC	
Recording in medical records	48 …. (59%)
KMC written internal protocols	46 …. (57%)
Specific training in the last 3 years	35 …. (43%)

Legend: *MV* mechanical ventilation, intubated infants, *NIV* noninvasive ventilation (CPAP, HFNC, etc.); ^a^: no answer or response such as “when the baby is stable”

### Characteristics of breastfeeding policies

Breastfeeding during KMC was encouraged in 70 (86%) neonatal units. In 64 (74%) units, the mother’s fresh breast milk was used, while in 24 (28%) units, breast milk expression at the side of the crib or incubator was supported, and 33 (38%) of the units had equipment for simultaneous bilateral breast milk expression. In 39 (45%) neonatal units, a milk bank was present. However, almost all centres (80, 93%) had a specific room for breast milk expression. Finally, we compared the duration of a single KMC session and the total daily KMC duration between 24-h-access NICUs and those with time-limited access. Although this comparison is not based on data within medical records but on the subjective evaluation of the staff in charge of completing the questionnaire, the duration of a single KMC session and the total daily KMC duration were reported to be longer in NICUs with 24-h access than in NICUs with time-limited access. The characteristics of breastfeeding policies are listed in the Table 4.

**Table 4:** Characteristics of breastfeeding policies

***Characteristics***	***Number and percentage of NICUs***
Promotion during KMC^a^	70 …. (86%)
Fresh milk use	64 …. (74%)
Milk expression at the crib side	24 …. (28%)
Simultaneous bilateral breast milk expression	33 …. (38%)
Milk bank	39 …. (45%)
Room for milk expression	80 …. (93%)

^a^number over 81 that promote KMC

## Discussion

This survey was performed in 2017, and although the regional response rate was heterogeneous, 80% of NICUs across Italy responded. Consequently, our analysis allowed us to provide an updated picture of current DC policies and to identify urgent needs for improving DC and KMC policies across Italian NICUs.

NICU open parental access is part of the organizational and structural practices suggested by the international recommendations for health and hospital policies to improve the care of hospitalized newborn babies, thereby enabling better clinical and neuropsychological development [14]. Over the last two decades, Italy has made significant strides towards allowing parents open access to NICUs. De Vonderweid and Leonessa [10] indicated from their 2001 survey that across 108 of 112 NICUs in Italy, only 29 and 24% of Italian NICUs provided open access for mothers and fathers, respectively. Similarly, in 2009, a study of eight European countries between 2004 and 2006 [9] reported open access in only 31% of NICUs in Italy and 27% in Spain. These reported access percentages were in stark contrast to the 100% of NICUs offering open access for both parents in Sweden, Denmark, and the UK; 90% in the Netherlands and Belgium; and 72% in France. In the vast majority of the units, especially after excluding NICUs from Italy and Spain, the durations of visits were not limited, and except for some limitations during medical rounds, visits were unrestricted [9]. Indeed, across all countries, medical rounds restricted access considerably more than other conditions. Only two countries within the European Union (EU), Spain and Italy, had time-limited open access policies for family members. Despite some improvements from 3 to 6 years prior (from 18 to 31% in Italy and from 11 to 27% in Spain), overall rates remained low [9].

The current survey has revealed significant progress, and the increase in persistently open units from 31 to 62% over a period of approximately 14–16 years is encouraging. Nevertheless, more work is needed to reach parity with northern European countries. In assessing whether parental access and attitudes towards parents are in accordance with a DC [15, 16] approach, our study shows that 39% of NICUs strongly limit entry times. Particularly striking is that we found that in 73% of partial-access NICUs, the average parental stay was 4 h, and further restrictions relating to shift changes, emergencies and medical rounds were reported.

Indeed, more than 64% of NICUs denied parents access to the ward during medical rounds. Another negative feature of Italian NICUs was the low percentage (45%) of units that provided access to relatives such as siblings, grandparents, uncles, and friends. We speculate that these negative aspects could be related to the fact that 70% of NICUs lacked accommodation facilities (sleeping room, family room, reading room, adjoining accommodation, dedicated kitchen). Indeed, our survey showed that a large number of NICUs do not provide a dedicated kitchen for parents or the option for a bed or accommodation near or inside the ward. Moreover, very few centres stated that they have family rooms.

The present survey showed that KMC is a well-known and widespread practice in all Italian regions; however, implementation strategies across centres were inconsistent and deviated from the WHO recommendations. The reported average time of a single KMC session and total daily KMC were 106 min and 166 min, respectively. This value falls within the minimum standard indicated by the WHO, which recommends that KMC should be performed as often as possible over 24 h, throughout the duration of the hospital stay, and at home after discharge from the hospital. Although KMC was promoted by most surveyed NICUs, restrictive policies regarding the entry of parents into the ward impede its practice. All the NICUs reported a clear (though not objectively quantifiable) relationship between access hours and the duration of KMC, where NICUs with 24-h open access allowed, on average, longer KMC sessions than NICUs with restricted access. In addition, KMC duration was further reduced in NICUs with especially restricted access times (less than 10 h per day) compared to those with access for more than 10 h per day. Limitations to KMC also reduce the opportunity for early breastfeeding [14, 17–19]. Our survey showed that 43% of Italian NICUs discontinue KMC early, either when the baby is transferred from the NICU to postintensive care (30%) or when the baby commences bottle feeding (13%).

Breastfeeding is usually favoured by a stable daily KMC practice [14, 19], especially for infants in intensive care. Unfortunately, the limited hours and additional restrictions for KMC across many Italian NICUs seemed to impede breastfeeding. Other factors that hindered breastfeeding in preterm infants included the lack of fresh breast milk from their mother (26%) and a lack of strategies for allowing preterm babies to breastfeed given an inability for the mother to express her breast milk at the crib side (72% of the centres) [20–23]. Mothers who can express their breast milk at the crib side can do so immediately after a KMC session, which is a natural stimulus for the oxytocin reflex [20–23]. Milk banks were present in only 45% of the units: the lack of donated human milk banks is another obstacle, as it leads to the commencement of formula milk feeding [23, 24]. Moreover, only 35% of the centres offered KMC at less than 29 weeks PMA; this figure may be due to the high percentage (26%) of vague answers, such as “when the child is stable” or similar responses. This statement does not allow the evaluation of these data with certainty; in addition, the frequent absence of shared protocols for the use of KMC and breastfeeding may ultimately limit both KMC and breastfeeding.

Strikingly, only 57% of units had written KMC protocols, with a significant gap across NICUs. This lack of written KMC protocols may indicate that staff do not perceive the presence of parents, KMC, and breastfeeding as legitimate therapeutic interventions. In contrast, evidence reported in the literature clearly stresses that empowering parents as primary caregivers, KMC, and breastfeeding are the strongest initiatives for promoting early attachment and interaction between the baby and family members [2, 6, 13–20, 24–31]. Conversely, early attachment and interaction are important early indicators for optimal child development [2, 6, 15, 17, 28, 30, 31]. Our survey outlines the need for the SIN to insist on residential courses to promote training on and the discussion, knowledge, and sharing of these concepts with NICU professionals. A survey approach, despite being difficult, complex, and time-consuming, is a vital tool for monitoring changes in DC-oriented policy throughout the country. This approach further allowed the identification of gaps and helped to uncover steps for overcoming the aforementioned restrictions. Our survey has clear limitations. Responses to a self-administered questionnaire may be subjective and reflect only the ideas of the person tasked with filling out the questionnaire and not those of the team or unit as whole. Actual inspection of the units would be ideal for checking individual unit policies and directly collecting the opinions of staff and parents. However, the actual inspection of such a large number of units throughout the country would be logistically difficult and expensive. Moreover, the subjectivity of team member responses and the lack of an accurate recording in the clinical charts of the number and length of KMC sessions are clear limitations to this study: in responses regarding KMC and breastfeeding, which are universally perceived as quality markers for the NICU, individual staff may overscore these aspects. We are planning to overcome this limitation in the next survey protocols, which will include the recording of activities with the possibility of building a “log-file” to collect measurable information, accepting the data on KMC duration only for those NICUs that perform accurate recording of the length and number of single KMC sessions.

Despite these limitations, an important strength of our survey is that the group who planned and oversaw this initiative was composed of 15 members from the DC study group board plus a number of regional contact persons who were responsible for relaying the questionnaire to individual NICUs across the 20 Italian regions. These members not only collaborated to encourage responses to the questionnaire (an 80% response rate among all Italian NICUs is a clear success) but also provided quality control given that they were familiar with individual NICUs and their specific features and protocols. Moreover, most of these experts have participated in the study since the initial discussions for the study plan and had the opportunity to see and discuss the results of the survey after the collection of the questionnaires.

## Conclusions

Since the early 2000s, the percentage of full 24-h access units in Italy has remarkably increased. However, facilities for family members remain inadequate for many centres. KMC has become a routine practice, but its frequency and duration still do not meet the WHO recommendations. As the implementation of DC and KMC is primarily a sociocultural issue, the SIN has identified education, teaching, and training in DC, KMC, and breastfeeding throughout the country as the primary SIN goals in upcoming years.

## Supplementary Information

**Additional file 1.** Questionnaire

## Data Availability

The questionnaires, in either paper or electronic format, and the first data review results were collected and saved by Dr. Claudia Artese and Dr. Silvia Perugi in the Careggi NICU of Florence University Hospital, Florence.

## Abbreviations

DC: developmental care
PMA: postmenstrual age
SIN: Italian Society of Neonatology
NDC: Neonatal Developmental Care
KMC: kangaroo mother care
NICU: neonatal intensive care unit
EU: European Union
CPAP: continuous positive airway pressure
MV: mechanical ventilation
HFNC: high-flow nasal cannula
