# Supplementary material for: Surveying family access: kangaroo mother care and breastfeeding policies across NICUs in Italy
Source: Ital J Pediatr. 2021 Dec 2;47:231. doi: 10.1186/s13052-021-01164-8 (PMC8638249; doi:10.1186/s13052-021-01164-8)
Supplement: Supplementary file 1 — Additional file 1. Questionnaire [file 13052_2021_1164_MOESM1_ESM.docx]

**4. Appendix: Questionnaire**

**Sections:**

1. **General characteristics of the NICUs**
2. **Access and attitude towards parents**
3. **Kangaroo Mother Care**
   1. **KMC operating procedures**
   2. **Breastfeeding support in the ward**
4. **General characteristics of the NICUs**
   1. Number of beds:
      1. intensive /_____/
      2. post-intensive /_____/
   2. Number of structured doctors on duty (including director) /_____/
   3. Number of nurses on duty (including nursing coordinator) /_____/
   4. Presence of the psychologist in NICU YES/NO

if YES

- - - - on request
      - routine (hours/week): ___/___
  1. Presence of the physiotherapist in the NICU YES/NO

if YES

- - - - on request
      - routine (hours/week): ___/___
  1. Presence of the NPI consultant or physiatrist in the NICU

if YES

- - - - on request
      - routine (hours/week): ___/___

1. **Access and attitude towards parents**
   1. mother's access
      - open
      - time-limited (maximum hours) /_____/
   2. father's access
      - open
      - time-limited (maximum hours) /_____/
   3. access of parents together
      - open
      - time-limited (maximum hours) /_____/
   4. access to other relatives
      - free
      - time-limited (maximum hours) /_____/
      - not allowed

if allowed, which relatives (tick):

- - - - siblings
      - grandparents
      - uncles
      - all
  1. Are the parents brought out during medical examinations (e.g. during rounds) and/or emergencies? YES/NO
  2. Which spaces among those listed below are available to parents
     - chair or armchair next to the child's crib
     - room for expressing milk
     - family room
     - reading room
     - dedicated kitchen
     - bed inside the ward
     - adjoining accommodation within the hospital
     - access to the hospital canteen
     - waiting room
  3. Periodic meetings with parents
     - monthly
     - fortnightly
     - weekly

1. **Kangaroo Mother Care**
   1. KMC operating procedures
      1. Is KMC practiced in the NICU? YES/NO

If YES

- - - - - 24 hours a day
        - for a limited number of hours /_____/
    1. Is KMC offered also to the father?

If YES

- - - - - 24 hours a day
        - for a limited number of hours /_____/
    1. How long does a daily session of KMC last on average? /_____/
    2. At what GA is KMC offered to parents on average? /_____/
    3. Is KMC practiced in all rooms of the ward? YES/NO

If not, in which room is it not practiced?

- - - - - NICU
        - Post-intensive intensive
        - neonatal pathology
    1. Is KMC offered several times a day? YES/NO
    2. Is KMC also offered to parents of twins? YES/NO

If YES, it is practiced

- - - - - routinely
        - occasionally
    1. Is KMC offered if the child is on ventilatory support? YES/NO

If YES, it is practiced

- - - - - routinely
        - occasionally

With which supports?

- - - - - VM
        - NCPAP
        - O_2_
        - high flows
    1. Is KMC offered if the child has a vascular catheter (CVC, CVO)? YES/NO

If YES, it is practiced

- - - - - routinely
        - occasionally
    1. Is the beginning and end of a KMC session noted? YES/NO

If YES, in which part of the medical record?

/_______________________________________________/

- - 1. During KMC, is early breastfeeding encouraged? YES/NO
    2. Usually when do you stop practicing KMC in the OU?
       - at discharge
       - in postintensive care unit
       - when the newborn starts feeding
    3. Have written protocols and/or recommendations on KMC been drawn up in the department? YES/NO
    4. Has there been any training in the department on KMC in the past three years? YES/NO
  1. Breastfeeding support in the ward
     1. Are mothers offered to breastfeed in the ward? YES/NO

If YES, where?

- - - - - in all hospital rooms
        - already in intensive care
        - only in some dedicated rooms

and when?

- - - - - often (with each visit)
        - rarely (once a day or less)
    1. Are there any restrictions on the use of fresh breast milk in the NICU? YES/NO

If so, which ones?

/____________________________________________________/

- - 1. Is simultaneous bilateral breast milk expression recommended? YES/NO
    2. Is expression recommended next to the child? YES/NO
    3. Is there a breast milk bank? YES/NO
    4. Is there a room where mothers can pump milk? YES/NO
    5. Are there breast pump available for mothers? YES/NO

If YES, how many? /______/


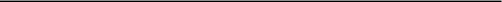


DEFINITIONS AND ABBREVIATIONS

KMC: Kangaroo mother care

UO: Complex operating unit with NICU and postintensive care

VM: mechanical ventilation

CVC: central venous catheter

UVC: umbilical venous catheter

GA: gestational age

NPI: infant neuropsychiatrist

CPAP continuous positive airway pressure

HFV

Name of the NICU: …………………………………………………………………………………

Address: .......................................................... Postal code………….City……………………

Landline...........................................................Mobile: ………………………………………….

e-mail: ………………………………………………………………………………………………

Name of the compiler: …………………………………………………………………………….

Signature of the medical director of the operating unit: ……………………………………………

Signature of the head nurse of the operating unit…………………………………………………
